# Social Media Policies in U.S. Medical Education: An Analysis of Content, Consistency, and Gaps

**DOI:** 10.7759/cureus.87134

**Published:** 2025-07-01

**Authors:** Elisheva Knopf, Leah Leidy, Darian Peters, George R Luck, Mario Jacomino

**Affiliations:** 1 Medicine, Charles E Schmidt College of Medicine, Florida Atlantic University, Boca Raton, USA; 2 Integrated Medical Science, Charles E Schmidt College of Medicine, Florida Atlantic University, Boca Raton, USA; 3 Women's and Children's Health, Charles E Schmidt College of Medicine, Florida Atlantic University, Boca Raton, USA

**Keywords:** education, medical school, medical student, policy, social media, technology

## Abstract

Background

Given the increasing prevalence and integration of social media platforms into various aspects of life, it is essential for medical schools to establish clear guidelines for their educational application and appropriate personal use. This study aims to assess the policies developed by medical schools in the United States and its territories addressing the use of social media.

Methods

Between June and July 2024, investigators examined the websites of all medical schools in the United States and its territories to assess their social media policies. The search involved reviewing student handbooks, policy webpages, and related websites. Specific search terms included "social media policy," "social networking policy," "social media," "social network," "social," and "media." Descriptive statistics, chi-squared analysis, and Fisher’s exact tests were utilized to describe and compare the categorical variables.

Results

Of the 199 U.S. medical schools, 166 (85%) had a social media policy (131 allopathic (M.D.) and 35 osteopathic (D.O.) schools). Among these policies, 98 (59%) were found in student handbooks, 45 (27%) on policy webpages, and 23 (14%) on other sites, typically related to technology and student affairs. Specifically, 131 (79%) policies were categorized as dedicated social media policies, while 35 (21%) were primarily included under student professionalism and technology policies. M.D. schools were more likely to have a social media policy than D.O. schools (p < 0.001). Schools in the South were also more likely to have social media policies (37% total policies, p < 0.001). Policy evaluation found that many policies mentioned platform names that are dated, such as Twitter instead of X, or failed to include the names of newer platforms, such as TikTok.

Conclusions

Most U.S. medical schools have social media policies; however, some need updates to reflect evolving platforms and current student use. Updating policies to address the evolving digital landscape will ensure that guidelines remain relevant and effective in promoting professionalism in medical education.

## Introduction

The past decade has witnessed a remarkable surge in the integration of social media platforms such as Facebook, Instagram, X (formerly known as Twitter), TikTok, and LinkedIn, transforming how people communicate, share information, and connect professionally. Social media usage has expanded across many sectors, including medical education, as these platforms become essential for accessing information, public engagement, and networking [1]. In medical education, it has been found that 86.1% of medical students in France actively learn via social media, spending two hours per day on YouTube, Instagram, and Facebook [2], while 61% of Jordanian students use social platforms for ≥3 hours daily, with 72.5% using them for academic content [3]. Moreover, 65% of residency applicants use social media to evaluate residency programs, nearly half reporting moderate to major influence on their perceptions of program selection [4]. In 2021, nearly 72% of adults in the United States were social media users, with the highest rates among those aged between 18 and 29, a demographic that includes many medical students and young professionals who use social media for both personal and professional purposes [5].

Healthcare professionals, including medical students, utilize social media to network, share medical knowledge, and engage with current research; however, this integration has risks. Privacy breaches, unprofessional behavior, and misinformation are significant concerns, underscoring the important need for guidelines on responsible use [6]. Medical students’ online conduct impacts their professional image and future careers; therefore, social media policies should clearly define expectations for digital professionalism to help guide medical students' online conduct [7].

In 2010, Kind et al. found that only 13 allopathic (M.D.) medical schools in the United States had formal social media policies, reflecting the early stage of institutional guidance on digital professionalism [8]. However, since then, the social media landscape has evolved dramatically, with new platforms and shifting norms. To remain relevant, social media policies must be updated regularly to reflect evolving digital norms [9].

Many medical institutions now integrate social media into educational programs to enhance learning and connect with wider audiences. Given the increasing role of social media in medical education and healthcare, it is vital to revisit and update social media policies in medical schools across the United States and its territories to ensure they align with current platforms and usage patterns [10]. In this descriptive study, we developed a conceptual framework that focuses on observation as described by Cook, Bordage, and Schmidt [11] to evaluate the prevalence, accessibility, and content specificity of publicly available social media policies across medical schools in the United States and its territories.

## Materials and methods

Data

We identified all M.D.-granting and osteopathic (D.O.-granting) medical schools in the United States and its territories from the American Association of Medical Colleges (AAMC) and American Association of Colleges of Osteopathic Medicine (AACOM) national websites, respectively [12, 13]. Only schools located within the United States and its territories (e.g., Puerto Rico, Hawaii) were included. Although the Liaison Committee on Medical Education (LCME) accredits some Canadian medical schools, those institutions were excluded from our analysis.

We mapped each school geographically according to the United States of America (USA) Consensus regions, Northeast, Midwest, South, and West, and we included Puerto Rico as part of the South and Hawaii as part of the West due to their close affiliation with those geographical regions [14]. We also collected the initial year of accreditation of each M.D. and D.O. school based on data provided on the LCME and AACOM websites, respectively [15, 16]. Accreditation is a formal process certifying that a medical school meets educational standards, marking the year an institution first qualified to offer a medical degree. By examining the year of each school’s initial accreditation, we aimed to analyze whether newer medical schools, those accredited more recently, are more likely to have implemented social media policies, as they may be more attuned to the evolving digital landscape. Furthermore, if social media policies are not being updated regularly, we hypothesized that older schools, accredited in earlier years, might be less likely to have such policies, reflecting the need for ongoing policy reviews across institutions. Some medical schools maintain campuses in multiple geographic regions. For consistency in regional analysis, we categorized schools based on the location of their primary campus as listed in the AAMC and AACOM directories.

We used the search function on each medical school website to identify the student handbook or policies webpage. If the policies were listed as separate sub-links, we examined each relevant site individually, including those for student professionalism, data use, and technology policies. Furthermore, if the student handbook or policy webpage could not be located, or if the search function was not available, we manually searched through the relevant websites of each school, including but not limited to academic practices, student affairs, medical education, information technology, and research pages. Finally, if we could not obtain information on policies from those websites or if the policy webpage required a student or university login, we contacted the medical schools by email or phone to ascertain if they had a public social media policy available. All policy searches and extractions were conducted by a team of three investigators (EK, LL, and DP). Discrepancies were discussed and resolved by consensus. Formal interrater reliability was not calculated, as the study was descriptive in nature, focusing on document presence and categorization. Our study does not encompass internal policies that are not shared online or policies that exist in a non-public format.

The handbooks and policy webpages were searched using the following keywords: “social media policy,” “social networking policy,” “social media,” “social network,” “social,” and “media.” From each medical school, we collected information on the presence of a social media policy, the policy’s location (whether in the student handbook, the policy webpage, or another webpage), and policy details. If a medical school mentioned social media on its website but did not have a specific policy, guidelines, or instructions for students on its use, those institutions were not counted as having a social media policy.

Statistical analysis

We used descriptive statistics to analyze policies by program type (M.D. vs. D.O.-granting schools) and geographic region of the United States. We also described characteristics of the social media policies amongst the subset of programs that had them available online. To evaluate associations between the categorical variables, we used a chi-squared analysis and Fisher’s exact test. These variables included the presence of a policy compared to program type (M.D. and D.O.), accessibility of the policy on the student handbook, policy webpages, or affiliated college of medicine website; whether the policy existed as a standalone social media policy or as part of another academic policy, the geographic region in America, and the timing of the medical school’s accreditation. All hypothesis tests were two-sided, and a P-value of < 0.05 indicated statistical significance. All statistics were generated using IBM SPSS Statistics version 29 (IBM Corp., Armonk, NY). 

## Results

Medical school policy breakdown 

We identified 199 medical schools, 158 (79%) of which were M.D. and 41 (21%) D.O. A student handbook was identified for 125 (63%) schools, and a policy webpage for 72 (36%) schools. For two schools (1%), both M.D., we did not find an online student handbook or policy webpage; therefore, we contacted both schools via email and received a response regarding their social media policy status. 

Social media policy presence by program and affiliation 

Of the 199 medical schools in the United States and its territories, a total of 166 (83%) medical schools had a social media policy. M.D. schools were more likely to have social media policies compared to D.O. schools (131 versus 35 policies) (p < 0.001). Policies were more likely to be obtained from the student handbooks (98, 59%) compared to the policy webpages (45, 27%) or other college of medicine-affiliated sites (23, 14%) (p < 0 .001). Similarly, social media policies were more likely to be their specific policy (131, 79%) as opposed to a subcategory within another policy (35, 21%) (p < 0.001) (Table 1). Furthermore, of the 33 schools that lacked a medical school social media policy, 10 had a social media policy under their university affiliation; however, we opted not to include these in our analysis since those policies were not specific for medical students.

**Table 1:** Medical schools, social media policy, and social media policy location M.D.: allopathic; D.O.: osteopathic; Percentages represent distributions within M.D. and D.O. schools with and without a social media policy; *Chi-square test utilized; **Represents statistical significance between categories, including the presence of social media policy amongst M.D. and D.O. schools; the presence of social media policy and social media policy location online; and presence of social media policy and policy classification.

Parameters	M.D. n, (%)	D.O. n, (%)	Test statistic*	p-value
Number of medical schools	158 (79)	41 (21)		
Presence of social media policy	131	35	χ² = 998.711	<0.001**
Social media policy location			χ² = 1996.000	<0.001**
Student handbook	70 (53)	28 (80)		
Policy webpage	40 (31)	5 (14)		
Other college of medicine-affiliated site	21 (16)	2 (6)		
Social media policy classification			χ² = 1933.174	<0.001**
Specific policy	106 (81)	25 (71)		
Subcategory of another policy	25 (19)	10 (29)		

Social media policy based on geographic region

Of the 166 medical schools with social media policies, they were most frequently found in schools located in the Southern states (61, 37%, p < 0.001). For those in the Midwest, Northeast, and Western states, there were 31 (19%), 41 (25%), and 33 (20%) medical schools with social media policies, respectively (Table 2).

**Table 2:** Social media policies by medical school geographic location Percentages represent distributions within the presence of social media policy to the geographic location in the United States; *Chi-square test utilized; **Represents statistical significance between the presence of social media policy and the geographic location in the United States

Presence of social media policy	Yes n, (%)	No n, (%)	Test statistic*	p-value
Number of medical schools	166 (83%)	33 (17%)	χ² = 1059.568	<0.001**
Midwest	31 (19)	9 (27)		
Northeast	41 (25)	2 (6)		
South	61 (37)	20 (61)		
West	33 (20)	2 (6)		

Social media policy based on the accreditation/establishment of a medical school

Of the 199 medical schools in the United States and its territories, we identified 142 that were accredited in the 20th century (124 M.D. and 18 D.O. schools) and 57 that were accredited in the 21st century (34 M.D. and 23 D.O. schools). We found 116 (82%) medical schools accredited in the 20th century to have social media policies, compared to the 50 (88%) medical schools accredited in the 21st century that had such policies (P = 0.56) (Table 3).

**Table 3:** Social media policies by medical school based on the year of initial accreditation Percentages represent distributions within the accreditation year of medical school, to the presence of social media policy within the medical school; *Chi-square test utilized; **Represents a comparison of associations between the presence of social media policy and the year of initial medical school accreditation

Presence of social media policy	Yes	No	Test statistic*	p-value
Year of medical school accreditation	n, (%)	n, (%)		
			χ² = 0.239	0.56**
20^th^ century (n = 142)	116 (82)	26 (18)		
21^st^ century (n = 57)	50 (88)	7 (12)		

Policy details 

**Figure 1:**
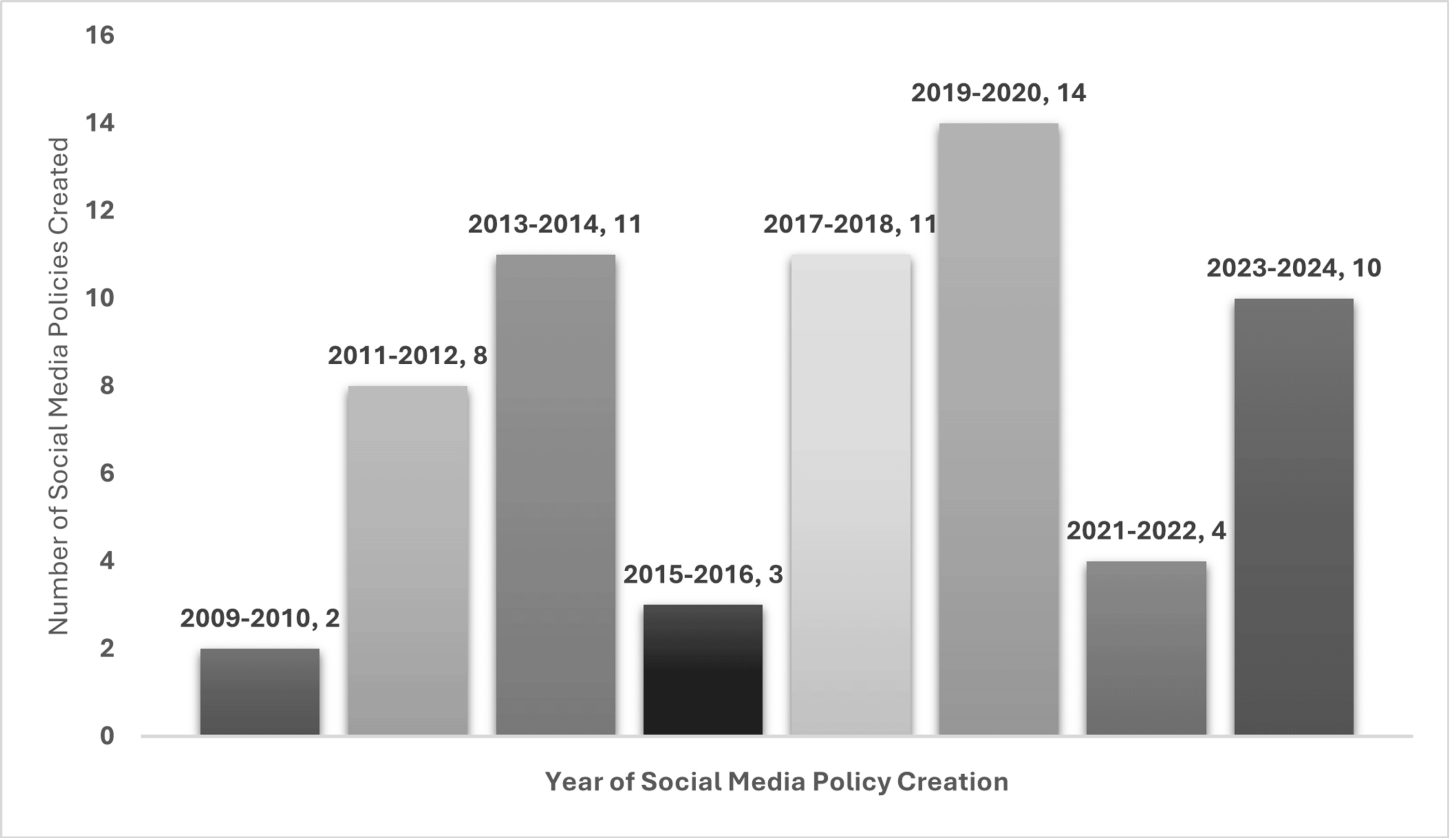
Number of social media policies created, grouped by the year of creation This bar chart illustrates the distribution of social media policies by their year of creation. The highest number of policies (14) were created during 2019–2020, followed by 11 policies each during 2013–2014 and 2017–2018. These data highlight the trends in institutional adoption of social media policies over time.

Of the 166 social media policies identified, 10 required a university or student login and were therefore inaccessible for review. Among the remaining 156 policies, 64 specified the year of creation. The most common years for policy creation were between 2013 and 2014 and 2017 and 2018, each with 11 policies (out of 64, 17%), and 2019 and 2020, with 14 policies (out of 64, 22%). The distribution of policy creation years is shown in Figure 1.

**Figure 2:**
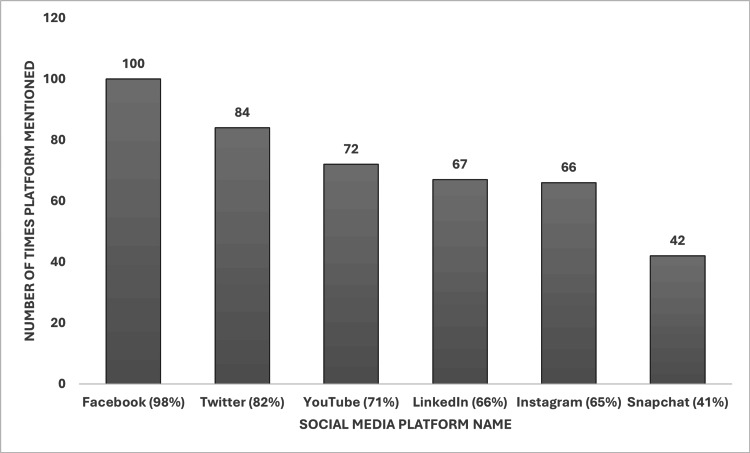
Specific social media platforms mentioned by social media policies This bar chart shows the frequency of mentions of specific social media platforms in institutional social media policies. Facebook was the most frequently mentioned platform (100 mentions, 98%), followed by Twitter (84 mentions, 82%), YouTube (72 mentions, 71%), LinkedIn (67 mentions, 66%), Instagram (66 mentions, 65%), and Snapchat (42 mentions, 41%). ‘X’ refers to the platform formerly known as Twitter.

We also found that 102 of the policies mentioned specific social media platforms. From these, the ones mentioned most frequently (with the % as a total of policies that mentioned specific platforms, n = 102) included Facebook (100 policies, 98%), Twitter (84 policies, 82%), LinkedIn (67 policies, 66%), Instagram (66 policies, 66%), YouTube (72 policies, 71%), and Snapchat (42 policies, 41%) (Figure 2). Only 20 policies included TikTok, and eight policies included Twitter’s new name, X. Other social media platforms that were mentioned less frequently included Flickr, Reddit, Wikis, Myspace, and Tumblr.

## Discussion

While the majority of United States medical schools and their territories have existing social media policies, our findings suggest that these policies may not be consistently updated or comprehensive enough to address recently emerging digital challenges. In our analysis, newer policies were typically more comprehensive than those from the 2010 analysis by Kind et al. [8], explicitly mentioning popular social media platforms such as Facebook, Twitter/X, and Instagram, providing guidelines for maintaining professionalism, and prioritizing patient confidentiality on public platforms. A survey conducted in the United States in 2022 found that 89.1% of medical students use social media websites, further eliciting the need for medical institutions to outline proper usage among students to maintain professionalism [17]. A similar study conducted in Turkey in 2015 revealed that the total social media usage among medical students was 93.4%, with 89.3% of medical students using social media specifically for professional purposes [18]. It has also been found that among medical students who use social media, guidance regarding proper social media usage among medical students is unclear [19]. This highlights the need to have established and accessible social media guidelines for medical students. While the increase in social media policies among medical schools indicates a positive trend toward adaptation, there remains room for improvement. Given the dual role of social media as both a public platform and an educational tool, medical schools should establish a structured policy review process that ensures social media guidelines are revised at least biennially.

Our findings reveal that M.D. institutions were significantly more likely to have formal social media policies compared to D.O. institutions. This difference does not appear to be driven by accrediting body requirements, as neither the LCME nor the Commission on Osteopathic College Accreditation currently mandates that medical schools maintain specific social media policies. Other potential contributing factors may include the age of the institution as well as differences in internal structure or emphases placed on professionalism and digital conduct within the curriculum.

While our study did not examine student enrollment data, differences in class sizes or campus structures between M.D. and D.O. programs may contribute to variations in policy development and visibility. For instance, D.O. programs often enroll large cohorts and may operate additional satellite campuses, likely complicating centralized policy implementation. This remains theoretical, and future research may explore whether institutional size or configuration influences the presence and accessibility of social media policies.

Additionally, policies should incorporate clear guidelines on topics such as patient privacy, misinformation, and professional standards and branding for medical students [6]. The failure to update policies not only reflects outdated terminology but also suggests a lag in institutional responsiveness to evolving digital landscapes. Without regular updates, policies risk becoming ineffective in guiding students on emerging ethical and professional challenges. For instance, TikTok, an increasingly popular platform in recent years amongst medical students [10], was mentioned in only 20 policies. 

Although it may be expected that newer medical schools would be more attuned to evolving digital platforms and more likely to implement formal social media policies, our findings did not reveal a significant difference based on accreditation year. One possible explanation is that older institutions have had more time to develop comprehensive policies, including updates to reflect the changing digital norms. Additionally, newer schools may be refining broader administrative structures and components related to curriculum, perhaps not yet prioritizing social media policy development. Such a point suggests that policy implementation may be driven less by institutional age and more by internal priorities and administrative focus.

The use of social media for personal use, educational purposes, professional development, and public health dissemination has increased significantly since its inception in the early 2000s. This increasing incorporation reflects the growing influence of social media in modern medical education and healthcare delivery. Programs now involve social media as a tool for teaching and learning, reflecting its importance in modern medical education. For example, the Mayo Clinic Academic Appointments and Promotions Committee began including social media and digital activities as a category in its criteria for academic achievement in 2016, prompting research into integrating social media into professional portfolios [20].

While using social media can offer benefits in terms of education and professional networking, it also requires one to tread lightly. One study conducted in 2009 found that 60% of medical schools in the United States reported incidents surrounding medical students posting unprofessional content on the internet. Some of the reported violations included but were not limited to breaches of patient confidentiality and posts using profane, sexually suggestive, or discriminatory language [21]. To ensure that policies regarding social media use are clear to both students and faculty per the guidelines listed in the school’s handbook, mandatory training on digital professional and ethical social media use, similar to current patient confidentiality and Health Insurance Portability and Accountability Act (HIPAA) training programs, can provide practical guidance on responsible digital engagement [20]. At present, there is no unified social media policy framework for medical schools in the United States and its territories. In light of this, we propose the creation of national guidelines, similar to those established by the American Medical Association, to ensure a consistent standard for medical students’ online presence nationwide. These guidelines should explicitly address critical issues such as patient confidentiality, professional conduct, and the prevention of online misinformation, safeguarding both medical students and patients. By implementing such a framework, we can rectify the current inconsistencies in content, enforcement, accountability, and overall efficacy across various institutions.

As future healthcare professionals in a world of increasingly expanding media, medical students must maintain their online professionalism. Social media can offer benefits in terms of education and professional networking but requires careful use to uphold the standards that come alongside a career as a physician. Their online behavior significantly reflects their professional image and can impact their careers. Furthermore, medical students represent their institution at a national and international level. The inappropriate use of social media can create a negative mark on the medical school’s image on public platforms. In turn, medical schools must implement formal policies that outline responsible social media use, ensuring medical students adhere to the professional standards set forth. Providing clear guidelines would allow medical schools to educate students regarding social media’s legal and ethical implications, promoting responsible digital communication. 

Study limitations

Several limitations affect the scope of our study. First, a major limitation was that our data was only obtained from publicly available information. This may have caused us to leave out policies that are internal or not available publicly online. Furthermore, the social media policies that we obtained may not reflect the most updated version of those policies. We also focused exclusively on policies that were created by medical schools for medical students. Although many medical schools require their students to abide by policies under their larger university system, we opted not to include these policies as they are not specific to medical student social media use. Policies that were located behind login portals or restricted to enrolled students were also excluded, even if we confirmed their existence. Our analysis was limited to publicly accessible policies specific to medical schools. Additionally, there is a point in time limitation to our study, as other schools may have policies in production or under review.

Finally, our study only encompasses social media policies provided by medical schools in the United States and its territories; however, the findings are relevant globally, and medical schools across the world could utilize the findings to address and update their social media policies. We also acknowledge that several medical schools maintain campuses across multiple geographic regions, and for consistency, our geographic classification was based on the primary campus location as listed in the AAMC and AACOM directories. This may introduce minor classification bias but was necessary to maintain uniform methodology.

Our study focused on policy presence, accessibility, and categorization but did not include a rubric-based assessment of quality or comprehensiveness. Further research exploring social media use and policies among medical students may wish to prioritize policy implementation, specifically assessing the extent to which medical students adhere to these guidelines. Identifying barriers to student compliance with social media policies and determining the best strategies for effectively distributing these policies may improve implementation outcomes. Medical schools without current policies or those seeking to enhance existing ones can draw insights from this study and established guidelines provided by the American Medical Association and American Osteopathic Association websites [22, 23]. Even though our study concentrates on medical students and their education, social media is available to students in other disciplines or careers within and outside the healthcare field. This descriptive study could therefore be extended to students in other disciplines, as each university or professional school develops and updates its social media policies.

## Conclusions

This study reveals that while the majority of U.S. medical schools have implemented social media policies, there is significant variability in their accessibility, specificity, and relevance to current digital platforms. Many policies are outdated, lacking references to newer platforms like TikTok, and not all offer practical guidance on managing the ethical and professional challenges that social media presents. Additionally, the location and classification of these policies vary widely, with some embedded deep within student handbooks and others only briefly mentioned under general professionalism guidelines. These inconsistencies may hinder students' ability to access and adhere to the standards expected of them. To address these gaps, medical schools should establish routine policy reviews, ensure their visibility and clarity, and incorporate targeted training on digital professionalism. Creating consistent, up-to-date, and actionable social media policies is critical to preparing future physicians for responsible engagement in an increasingly digital healthcare environment.
